# Veterinary Medicine

**Published:** 1847-07

**Authors:** 


### VETERINARY MEDICINE.

Dr. Brooks, of Boston, is preparing, in Paris, a work on veterinary medicine, which he expects to issue during the present year. Our correspondent there says Dr. B. has favored him with an inspection of the manuscript, from which he feels authorized to promise that the performance will be a creditable one. In France and England this subject commands the attention and respect of the profession; with us it has hitherto been neglected, but the forthcoming work of Dr. B. will probably induce us to bestow upon it some share of regard.

